# Medical student fitness to practise committees at UK medical schools

**DOI:** 10.1186/1756-0500-2-97

**Published:** 2009-06-06

**Authors:** Jocelyne Aldridge, Sally A Bray, Timothy J David

**Affiliations:** 1 Medical Schools Council, Woburn House, Tavistock Square, London, WC1H 9HD, UK; 2 Faculty of Medical and Human Sciences, University of Manchester, Simon Building Room 4.34, Oxford Road, Manchester, M13 9PT, UK

## Abstract

**Background:**

The aim was to explore the structures for managing student fitness to practise hearings in medical schools in the UK. We surveyed by email the named fitness to practise leads of all full members of the UK Medical Schools Council with a medical undergraduate programme. We asked whether student fitness to practise cases were considered by a committee/panel dedicated to medicine, or by one which also considered other undergraduate health and social care students.

**Findings:**

All 31 medical schools responded. 19 medical schools had a fitness to practise committee dealing with medical students only. Three had a committee that dealt with students of medicine and dentistry. One had a committee that dealt with students of medicine and veterinary medicine. Eight had a committee that dealt with students of medicine and two or more other programmes, such as dentistry, nursing, midwifery, physiotherapy, dietetics, social work, pharmacy, psychology, audiology, speech therapy, operating department practice, veterinary medicine and education.

**Conclusion:**

All 31 UK medical schools with undergraduate programmes have a fitness to practise committee to deal with students whose behaviour has given rise to concern about their fitness to practise. The variation in governance structures for student fitness to practise committees/panels can in part be explained by variations in University structures and the extent to which Universities co-manage undergraduate medicine with other courses.

## Background

The General Medical Education and Registration Council of the United Kingdom (shortened to General Medical Council in 1951) was created by the Medical Act of 1858 [1] and charged with establishing a register "to distinguish the qualified from the unqualified" [2]. The General Medical Council established a medical student register, which was put "on hold" during the second world war and never reactivated [2].

The document "Tomorrow's Doctors", first published by the General Medical Council in 1993, and revised in 2003 [3], provided recommendations for medical schools which identified the knowledge, skills, attitudes and behaviour expected of new graduates. There was emphasis on the need to protect patient safety (paragraph 83), and a clear statement that by awarding a medical degree, a university is confirming that the graduate is fit to practise (paragraph 84).

In 1997, the General Medical Council published its first guidance for medical students entitled "Student Health and Conduct" [4]. This document, which has been superseded and no longer reflects current policy, included advice on specific student issues such as anxiety and stress, psychiatric illness, drug and alcohol abuse, behavioural problems and physical illness, as well as addressing general topics such as the length of the course (a maximum of seven years was recommended), the duty to protect patients, the need for confidentiality, and the options for students "not suited to medicine". In addition, since, 2005, graduating students have, when applying for General Medical Council registration, been required to confirm that they are fit to practise.

In July 2001, Eversheds produced a report to Universities UK and the Council of Heads of Medical Schools on medical student fitness to practise, and recommended principles for the adoption by universities of fitness to practise procedures "that are fair to individuals, reflecting legislative requirements, and that do not necessarily follow a uniform and rigid pattern across all institutions. Subject to consistency with the principles, the detailed procedures will be for determination by individual institutions taking account of their own statutory and regulatory systems" [5]. Those universities that had not established fitness to practise arrangements were urged to do so as a matter of priority. In September 2007, the Medical Schools Council and the General Medical Council published their detailed guidance on matters relating to fitness to practise in medical students [6]. This set out the professional behaviour expected of medical students, following the headings used in the General Medical Council's guidance for doctors "Good Medical Practice" [7]. It also delineated matters relating to medical student fitness to practise, such as areas of misconduct, sanctions, threshold of acceptable behaviour, making decisions, and key elements in student fitness to practise arrangements. The application of this guidance in medical schools, along with the recent implementation of General Medical Council training in fitness to practise procedures for medical school staff, promotes a consistent approach to student fitness to practise in a diverse group of institutions.

Student fitness to practise is also being addressed by other healthcare professions, and, for example, in August 2007, the Nursing and Midwifery Council published their guidance "Good health and good character. Guidance for educational institutions" which was intended to ensure consistency about how the Nursing and Midwifery Council's requirements were interpreted and put into practice [8]. This guidance also set out a requirement that educational programme providers should take appropriate action if any issues relating to good health or good character arise, and it stated that from September 2007 all programme providers were required to have a fitness to practise panel to consider health and character issues and ensure that public protection was maintained. This guidance was revised in June 2008 [9].

A national workshop for medical school administrators on the subject of medical student fitness to practise, run jointly by the University of Manchester (Tim David and Sally Bray) and Field Fisher Waterhouse (Sarah Ellson and Judith Chrystie), a firm of lawyers specialising in professional regulation, was held at the University of Manchester on 8 November 2007, a few months after the publication of the Medical Schools Council-General Medical Council guidance. At this workshop it became apparent that there was variation in the way that student fitness to practise panel hearings were managed, reflecting the different university statutes and regulations. In December 2008, Tim David contacted Jocelyn Aldridge at the Medical Schools Council, to enquire about the arrangement for student fitness to practise cases, and to assist with this enquiry a survey was conducted to examine this variation, and this report documents the findings.

## Methods

On 16 December 2008, Jocelyne Aldridge at the Medical Schools Council emailed the named fitness to practise leads of all 31 full members of the Medical Schools Council with a medical undergraduate programme, to ask whether there was a dedicated fitness to practise committee just for medicine or a pan-faculty arrangement for fitness to practise so that students from other disciplines were considered by the same committee. All 31 responded.

## Results

19 medical schools had a fitness to practise committee dedicated to medical students. Three had a committee that dealt with students of medicine and dentistry. One had a committee that dealt with students of medicine and veterinary medicine. Eight had a committee that dealt with students of medicine and two or more other programmes, such as dentistry, nursing, midwifery, physiotherapy, dietetics, social work, pharmacy, psychology, audiology, speech therapy, operating department practice, veterinary medicine and education. Some schools with a fitness to practise committee dedicated to medicine nevertheless used regulations which were shared with one or more other programmes.

## Discussion

The General Medical Council and Medical Schools Council have been engaged in joint working on student fitness to practise since 2000. In 2005, the General Medical Council and the Medical Schools Council established the joint Student Fitness to Practise Working Group, which published the first edition of its guidance for medical schools and medical students in 2007 [6]. A revised version of the guidance has since been developed by the working group and was published on 9 March 2009 [10]. This advice was advisory rather than mandatory, but it was pointed out that General Medical Council quality assurance reports on medical schools may recommend that they comply with the guidance. Given that the General Medical Council has to be satisfied that graduates applying for registration are fit to practise, the guidance said that "it would be surprising if a medical school thought it sensible to disregard this guidance" [6]. The guidance advised that medical schools should issue fitness to practise policy documents which amongst various matters should describe the procedures to be applied to students. The data from this survey shows that by the end of 2008, all 31 UK medical schools with undergraduate programmes had a fitness to practise committee to deal with medical students.

Whilst a fitness to practise committee dedicated to medical students was the most common model (19 medical schools), it was also common for a student fitness to practise committee to deal with one or more (range one to nine) other courses (12 medical schools). This variation may in part be explained by variations in the nature and extent to which University structures co-manage undergraduate medicine with other courses. It may also be the result of different philosophies and working patterns.

An additional source of variation is that each university has its own regulations, both general university regulations (for example covering attendance, misconduct, dress code, and drugs and alcohol use) and specific regulations for each programme, and the multiplicity of local regulations and procedures makes it more difficult to harmonise arrangements between healthcare programmes or between different universities.

It is believed that the numbers of medical students who are irretrievably unsuitable for a career in medicine are very low [11,12]. But it is evident that these extreme cases are but the tip of an iceberg of students whose health or behaviour cause concern about their fitness to practise, as exemplified, for example, by individual published case reports [13-15]. Plainly institutions delivering healthcare education must have systems in place to educate students about professionalism [16], and to deal with problem individuals.

## Conclusion

The data obtained in this study indicate that by the end of 2008, all 31 UK medical schools with undergraduate programmes had established a committee to deal with students whose health or behaviour has given rise to concern about their fitness to practise. Medical schools, with the support of the General Medical Council and the Medical Schools Council, continue to work to develop greater consistency in their approach to student fitness to practise, and most recently, on 3 November 2008, the General Medical Council commenced a series of training events around the UK for staff involved in implementing medical student fitness to practise procedures.

## Competing interests

The authors declare that they have no competing interests.

## Authors' contributions

TD asked the question which formed the basis for JA emailing all UK medical schools and collecting data from the responses. TD produced a first draft, which was further developed and finally approved by all the authors.
